# Diversity of Water Yam (*Dioscorea alata* L.) Accessions from Côte d’Ivoire Based on SNP Markers and Agronomic Traits

**DOI:** 10.3390/plants10122562

**Published:** 2021-11-24

**Authors:** Lassana Bakayoko, Désiré N’Da Pokou, Abou Bakari Kouassi, Paterne A. Agre, Amani Michel Kouakou, Konan Evrard Brice Dibi, Boni Nzue, Jean M. Mondo, Patrick Adebola, Oluyemi T. Akintayo, Asrat Asfaw, Assanvo Simon Pierre N’Guetta

**Affiliations:** 1 UFR Biosciences, Université Félix Houphouët Boigny, Abidjan 582, Côte d’Ivoire; Lassana06bakayoko@gmail.com (L.B.); abou_kouassi@yahoo.fr (A.B.K.); nguettaewatty@yahoo.fr (A.S.P.N.); 2 Laboratoire Central de Biotechnologie (LCB), Centre National de Recherche Agronomique (CNRA), Abidjan 1740, Côte d’Ivoire; desire.pokou@gmail.com; 3 International Institute of Tropical Agriculture (IITA), Ibadan 5320, Nigeria; m.mubalama@cgiar.org (J.M.M.); a.amele@cgiar.org (A.A.); 4 Station de Recherche sur les Cultures Vivrières (SRCV), Centre National de Recherche Agronomique (CNRA), Bouaké 633, Côte d’Ivoire; amanimichelkouakou@gmail.com (A.M.K.); dibikonan@yahoo.fr (K.E.B.D.); bnzue1@gmail.com (B.N.); 5 Department of Crop Production, Université Evangélique en Afrique (UEA), Bukavu 3323, Democratic Republic of the Congo; 6 International Institute of Tropical Agriculture (IITA), Abuja 901101, Nigeria; p.adebola@cgiar.org; 7 École Supérieure d’Agronomie, Université de Lomé, Lomé 1515, Togo; akintayooluyemi@gmail.com

**Keywords:** *Dioscorea alata*, genetic diversity, molecular markers, DArT-seq, population structure, agronomic trait

## Abstract

*Dioscorea alata* (L.), also referred to as water, winged, or greater yam, is one of the most economically important staple food crops in tropical and subtropical areas. In Côte d’Ivoire, it represents, along with other yam species, the largest food crop and significantly contributes to food security. However, studies focusing on better understanding the structure and extent of genetic diversity among *D. alata* accessions, using molecular and phenotypic traits, are limited. This study was, therefore, conducted to assess the pattern of genetic variability in a set of 188 *D. alata* accessions from the National Agronomic Research Centre (CNRA) genebank using 11,722 SNP markers (generated by the Diversity Arrays Technology) and nine agronomic traits. Phylogenetic analyses using hierarchical clustering, admixture, kinship, and Discriminant analysis of principal component (DAPC) all assigned the accessions into four main clusters. Genetic diversity assessment using molecular-based SNP markers showed a high proportion of polymorphic SNPs (87.81%). The analysis of molecular variance (AMOVA) showed low molecular variability within genetic groups. In addition, the agronomic traits evaluated for two years in field conditions showed a high heritability and high variability among *D. alata* accessions. This study provides insights into the genetic diversity among accessions in the CNRA genebank and opens an avenue for sustainable resource management and the identification of promising parental clones for water yam breeding programs in Côte d’Ivoire.

## 1. Introduction

*Dioscorea alata* (L.) yam, commonly known as water yam, winged yam, or greater yam, is one of the most widespread root and tuber crops in tropical and subtropical areas of the world. It is produced mainly for its starchy underground tubers [1,2]. Water yam was introduced into Central and West African countries, probably from the southeast Asian–Oceanian regions, during the 16th century [3,4]. *Dioscorea alata* represents, along with other yam species, such as *D. rotundata* (Poir.) and *D. cayenensis* (Lam.), the second most important edible root and tuber crop after cassava in West Africa, where yam plays an essential role in the nutrition, food security, income generation, and socio-cultural lives of more than 300 million people [5]. In West and Central Africa, yam is extensively produced in the African yam belt, a region including countries such as Nigeria, Ghana, Côte d’Ivoire (Ivory Coast), Benin, Togo, and Cameroon [5,6,7].

In Côte d’Ivoire, yam is the most economically important food crop and is mainly grown in the central and northern savannah areas [8,9,10]. Among grown yam species, *D. alata* is dominant and accounts for ~60–70% of the total yam production [10,11]. In 2018, Côte d’Ivoire ranked as the third-largest yam producing country in the world after Nigeria and Ghana, with a cumulative yam production of 7.25 million tons [10]. The massive adoption of *D. alata* cultivation in the country stems from many factors, including ease of propagation, rapid growth allowing it to suppress weeds, stable yield under ranges of soil fertility and ecologies, long post-harvest tuber storage, and high nutritional values for human consumption and livestock feed [11,12,13,14].

Despite the importance of water yam as a staple food, youth employment, and income generation, many biotic and abiotic constraints threaten its production worldwide [14,15]. Yam production has mainly relied on the rapid increase in cultivated acreage rather than increased productivity per unit area, a farming system that encourages deforestation and degradation of natural resources [16]. For the conservation of natural reserves, such as forests and savannah, and sustainable management of yam genetic resources, there is a need to provide farmers with improved varieties from diverse genetic backgrounds that combine high yields with pest and disease resistance and acceptable tuber quality [17]. Such an approach requires a thorough understanding of the genetic diversity within the existing germplasm for the effective selection of parental clones for hybridization [18].

Phylogenetic relationships of water yam in Côte d’Ivoire have not been well established among yam accessions because of limited morphological markers and flow of planting materials across areas (as a result of trade and informal exchange of planting materials) [9]. Consequently, several cultivars that have the same names or different names are allocated to a single cultivar, depending on local languages (ethnic groups) and locations [17]. As earlier stated, the assessment of the pattern of genetic variability within the existing yam germplasm will be essential to identify genotypes possessing genes/traits of interest and that can be rationally used to develop new varieties [19]. It is, therefore, necessary to carry out a thorough characterization of the genetic resources of *D. alata* in Côte d’Ivoire to allow the sustainable and effective exploitation and management of existing diversity for breeding purposes to produce improved varieties [18,20]. For more refined genetic diversity studies, using both molecular and phenotypic data have been advocated to control drawbacks of both approaches [2,18].

Several Next-Generation Sequencing (NGS) techniques, including Genotyping-by-Sequencing (GBS) and Diversity Array Technology (DArT), have recently been developed. They have enabled the discovery and generation of genome-wide high-throughput markers, such as SNPs, for genetic mapping, quantitative trait loci (QTL) identification, and molecular plant breeding and genetic diversity studies in yam [2,14,18,21,22,23,24]. However, little has been done to include molecular marker systems in the genetic diversity studies of *D. alata* accessions in Côte d’Ivoire, and existing information is still based on morphological data and marker systems with low reproducibility.

The main objective of the present study was to assess the genetic structure and diversity pattern of the in vivo collection of *D. alata* accessions of the National Agronomic Research Centre (CNRA) using SNP molecular-based DNA markers and nine agronomic descriptors to update the information on the yam genetic diversity in Côte d’Ivoire. The results should provide research institutes and plant breeding programs with reliable data for the choice of plant materials to be used for the creation and selection of new varieties that are more productive and resistant to diseases, pests, and environmental constraints and meet growers and consumers’ tuber quality needs.

## 2. Results

### 2.1. Variability in Agronomic Traits of Assessed Genotypes

Multiple analysis of variance (MANOVA) based on Wilks’ lambda test revealed highly significant differences (F = 0.175; *p* < 0.001) among accessions for agronomic traits across years. The Fisher least significant difference (LSD) test showed that the variances for all traits were significantly different (*p* < 0.05) between 2018 and 2019 (Table 1). High tuber yield (8.71 t ha^−1^) and plant vigor were recorded in 2018 compared to 2019 (7.43 t ha^−1^). In contrast, anthracnose disease severity score (ADSS), tuber length, tuber width, tuber circumference, dry matter content, and weak vigorous plant rate (WVPR) were higher in 2019 than they were in 2018 (Table 1).

### 2.2. Genotype and Genotype × Year Interaction Effects on Agronomic Traits of D. alata Accessions

The analysis of variance (ANOVA) revealed that the agronomic traits of *D. alata* accessions were significantly influenced by the genotype effects except for the medium vigorous plant rate (MVPR). The variances across the years showed highly significant differences among genotypes for all agronomic traits. However, the genotype × year interactions indicated highly significant differences among genotypes for five of the nine traits evaluated, including anthracnose disease severity (ADSS), tuber length, tuber width, tuber circumference, and rate of weak vigorous plants (WVPR). The interaction effects were not significant for tuber yield, vigorous plant rate, medium vigorous plant rate (MVPR), and dry matter content. In addition, mean square values of genotypes were higher than that of the genotype × year interaction for all agronomic traits (Table 2). The broad-sense heritability for all agronomic traits was highly significant (H^2^ > 0.50). The highest value (H^2^ = 0.80) of broad-sense heritability was obtained with VPR, while tuber length had the lowest H^2^ value (0.56).

### 2.3. Population Structure Analysis and Genetic Diversity Pattern in 188 Accessions of D. alata Using Molecular-Based SNP Markers

A total of 45,012 SNP markers from 188 *D. alata* accessions were generated by the Diversity Arrays Technology (DArT) platform. The transformation of these allelic sequences into genotypic data resulted in a raw data file of 22,506 SNPs, and after quality control analysis (SNP filtering), only 11,722 high-quality SNPs were retained for further analyses.

**Figure 1:**
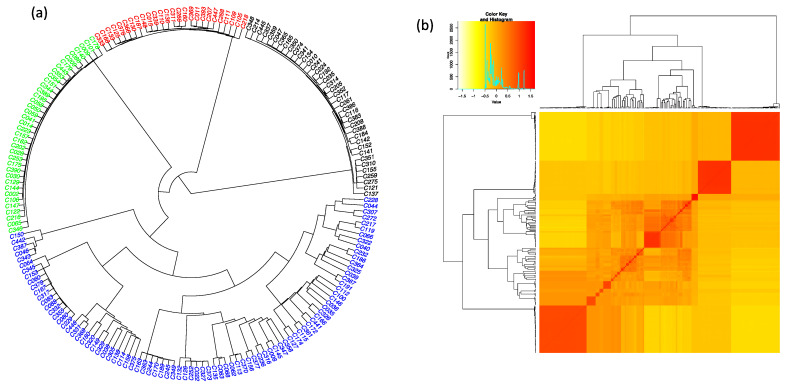
Genetic relationship among 188 accessions of *D. alata* based on 11,722 SNPs: (**a**) Hierarchical circular clustering dendrogram generated using the UPGMA method and Jaccard’s dissimilarity matrix. Different colors indicate different groups identified: Cluster 1 (red), Cluster 2 (blue), Cluster 3 (black), and Cluster 4 (green), (**b**) Kinship Heatmap; the color gradient shows the similarity among accessions.

Clustering analysis using Jaccard’s dissimilarity matrix and the Unweighted Pair-Group Method with arithmetic Average (UPGMA) methods assigned the 188 accessions into four main genotypic clusters (Figure 1a). Kinship analysis also indicated the presence of a population stratification based on the proportion of shared alleles and properly distinguished four main groups as suggested by clustering analysis (Figure 1b). The two analyses (hierarchical clustering and kinship) all assigned the accessions into different clusters irrespective of their own varietal groups. All variety groups were present in the four clusters identified. Through phylogenic analysis, cluster one was the smallest group, only accounting for 26 accessions (13.83%) and essentially made up of the ‘Florido’ variety group and derived hybrid lines. Cluster 2 was the largest; its membership had 81 accessions (43.08%), which mainly consisted of ‘Betebete’, ‘Florido’, and derived hybrid lines. Clusters 3 and four comprised 43 and 38 accessions, respectively. The high proportions of their memberships were made of the ‘Betebete’ and derived hybrid lines (Figure S1). However, the agronomic performance of each molecular cluster based on multiple analysis of variance revealed that the variations among accessions within clusters were insignificant according to Wilks’ lambda test (F = 0.90; *p* = 0.61) for all assessed traits (Table S1).

**Figure 2:**
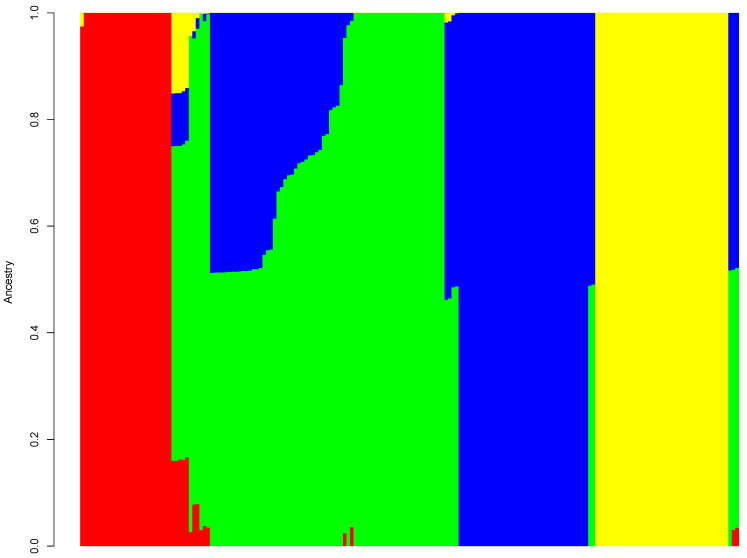
Grouping pattern in 188 *D. alata* accessions at *K* = 4 based on the Bayesian clustering method. The color displays each cluster: Red (cluster 1), Green (cluster 2), Blue (cluster 3), Yellow (cluster 4). Each vertical bar corresponds to an accession, and the color proportion in each bar represents the probability of each accession being affiliated to the different clusters.

Population analysis based on cross-validation suggested four clusters as the optimum number of genetic groups within the accessions. Through the Admixture analysis, most of the accessions were properly assigned to a genetic group, and only a few were considered as admixt (Figure 2). The membership probabilities for assigning affiliation to accessions varied from 0.51 to 1. Out of the 188 accessions, only three accessions (CIVCDA 252, CIVCDA 236, and CIVCDA 316) were defined as admixt using an ancestry cut-off of 50%. Across clusters, the largest value of membership probability was 0.999. Clusters 1 and 4 consisted of accessions that were genetically distinct from those in the two other clusters, with the lowest value and membership probability of 0.974 and 0.999, respectively. On the other hand, the smallest values of membership probability for accessions in clusters 2 and 3 were 0.512 and 0.509, respectively. In line with understanding the population structure, the admixture was plotted at K = 2, 3, 5, 6, 7, and 8 (Supplementary Figure S2). Considering the membership probability at K = 2 and K = 3, all the accessions were 100% assigned to their respective groups (Figure S2A,B). At K = 5, 6, 7, and 8, few accessions were identified as admixt (Figure S2C–F).

**Figure 3:**
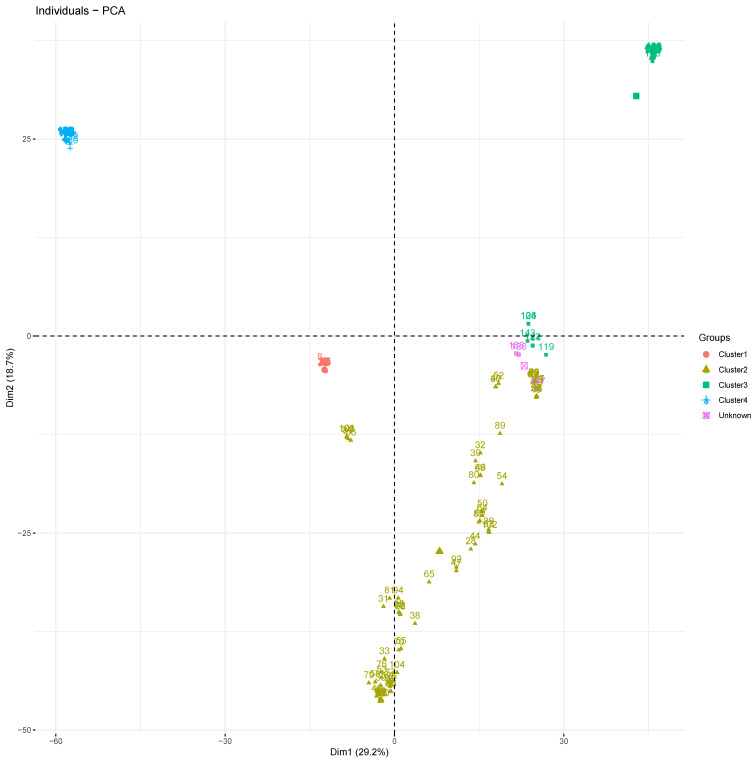
Principal component plot showing clustering of the 188 *D. alata* accessions into four clusters. Each color represents a cluster: cluster 1 (red), cluster 2 (gold), cluster 3 (green), cluster 4 (blue), and admixture (purple).

Through principal component analysis (PCA), the first and second components explained only 29.2 and 18.7% of the total variance, respectively, and account for 47.90% of the total observed variation (Figure 3). The analysis confirmed the clustering of accessions into the groups suggested by the admixture method. All accessions in each cluster were grouped together, and accessions in cluster 2 seemed more heterogeneous. The accessions considered to be admixtures were also clustered together and identified as an unknown group by PCA analysis. According to the first two components, accessions in clusters 2 and 3 should be divided into three main subgroups each (Figure 3).

### 2.4. Discriminant Analysis of Principal Components (DAPC)

**Figure 4:**
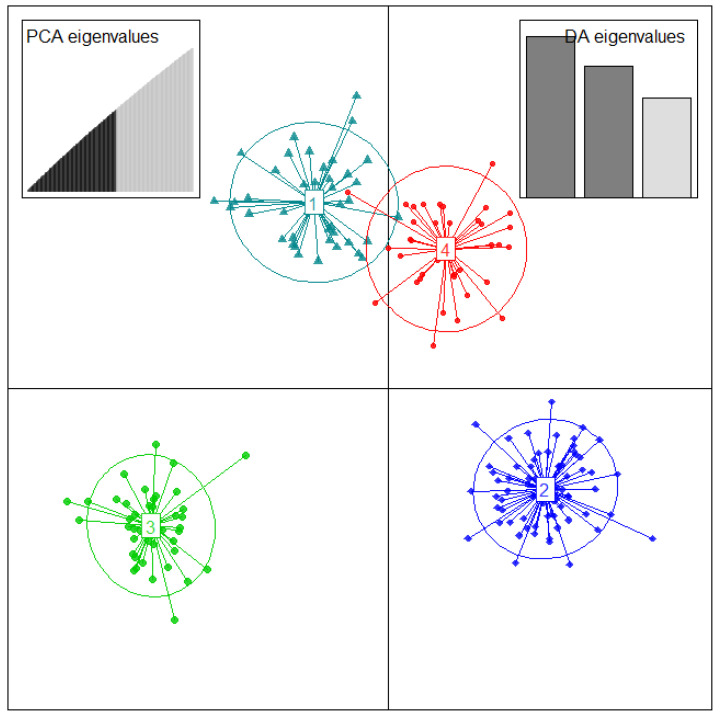
Discriminant analysis of principal components based on 11,722 SNPs and the allelic sequences of 188 *D. alata* genotyped accessions from in vivo CNRA yam genebank. The axes represent the first two linear discriminant functions (LD). The circles correspond to each cluster identified. The dots in each circle represent individuals, and the number in the circle center indicates different subpopulations identified by DAPC analysis.

DAPC analysis and based on the Bayesian Information Criteria (BIC) detected a maximum of K = 4 to be retained with clearly distinct clusters (Figure S1 and Figure 4).

The membership probability of each accession to be assigned into different clusters was 100% for all accessions, and no admixture or accession with multiple affiliations was detected by DAPC analysis. Cluster membership estimates showed that cluster 2 accounted for the largest number of accessions (69), followed by cluster 3 with 42 accessions, cluster 1 (41 accessions), and cluster 4 was found to be the smallest group with 36 accessions (Figure 4).

Multiple analysis of variance showed highly significant differences (F = 2.93; *p* < 0.001) between the agronomic performances of clusters defined according to Wilks’ lambda test. The Fisher LSD test carried out following an analysis of variance for each of the quantitative traits revealed that cluster 1 consisted of accessions with the highest agronomic performance, including high yield (7.17 t ha^−1^), high resistance to anthracnose disease (2.77), high dry matter content (28.65%), and the production of tubers with the highest circumference (23.25 cm), length (17.88 cm), and width (10.47 cm) (Table 3).

However, the weak vigorous plant rate (WVPR) was the lowest at 18.11% (Table 3). This cluster contained all variety groups with a dominance of hybrid lines (29.27%), followed by ‘Betebete’ (21.95%) and ‘Douoble’ (14.63%). Cluster 3, predominantly made up of ‘Betebete’ (30.95%) and related hybrid lines (26.19%), was identified as the cluster with the lowest agronomic performance. Accessions in this cluster were more susceptible to anthracnose disease, with the highest value of disease severity score (2.91) and weak vigorous plant rate (28.02%).

It is noteworthy that the phylogenic tree and DAPC analysis only affiliated 48 accessions (25.53% of the total number of accessions) into the same clusters.

**Figure 5:**
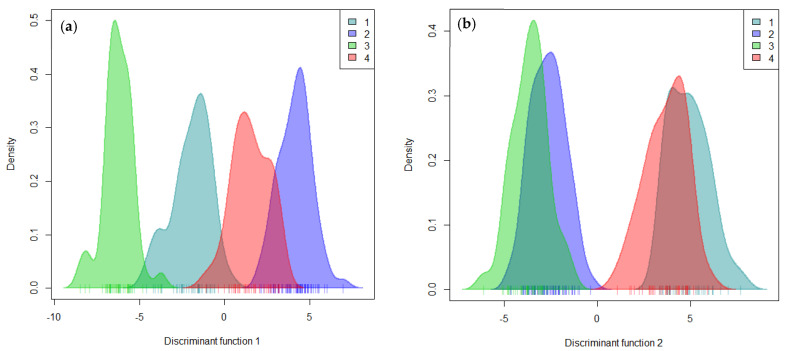
Characterization of grouping patterns using the first two linear discriminant functions in DAPC analysis: (**a**) linear discriminant function 1; (**b**) linear discriminant function 2.

The first linear discriminant function (LD1) clearly separated clusters 1 and 3 from clusters 2 and 4. Clusters 1 and 3 were presented in the negative area of LD1, while clusters 2 and 4 were located in the positive area (Figure 5a). The second linear discriminant function (LD2) distinguished clusters 1 and 4 (positive side) from clusters 2 and 3 (negative side) (Figure 5b). However, a comparison of each cluster membership through the four clustering methods revealed that the assignment of accessions into different groups identified was contrasting. Cluster membership for hierarchical clustering analysis was in perfect alignment with kinship membership and Bayesian-based clustering methods.

### 2.5. Genetic Diversity within Groups and Population Using Molecular-Based SNP Data

The average of polymorphic SNP markers was 87.81%. It was highest in cluster 2 with 99.98% and lowest in cluster 1 with 80.47%. A total of 23,444 alleles was detected, with an average of two alleles per locus. Private alleles (175) were only detected in cluster 2, and none of the clusters contained all the identified alleles. Cluster 2, with the largest number of alleles, only accounted for 23,442 alleles, representing 99.99%. The average number of alleles per locus in each cluster ranged from 1.81 (cluster 1) to 2 (cluster 2). The Shannon’s information index, which measures the diversity pattern within a population, was generally low (<0.50) in three clusters with an average of 0.34 (Table 4). Cluster 2, with the largest Shannon’s information index (0.54), appeared as the most diversified group. Observed heterozygosity in the total population was 0.294 and varied within the clusters from 0.185 to 0.259, whereas expected heterozygosity ranged from 0.096 to 0.278, with a value of 0.158 in the total population. The inbreeding coefficient was very low (Fis < 0) in the population and three clusters. The Fis value was −0.398 in the population and −0.927, −0.529, and −0.876 in clusters 4, 3, and 1, respectively. However, in cluster 2, the value of Fis was positive (0.068). Minor allele frequencies were 0.209 in the total population. In each cluster, the values ranged from 0.093 (cluster 1) to 0.202 (cluster 2). The groups of accessions, as well as the total population, recorded missing values with frequencies less than 0.05. The difference between Ho and He estimated using the Mann–Whitney U test was significant (p < 0.05) in all groups and the entire population. Of the four groups, the average rank of observed heterozygosity was greater than that of the expected one in three groups, and it was only in cluster 2 where the mean rank of H_e_ was higher than that of H_o_ (Table 4).

The Hardy–Weinberg Chi^2^ equilibrium test indicated that 37.82% of the markers were not able to make differences (*p* > 0.05) between the observed and expected heterozygosity, while 62.18% showed significant differences. Clusters 1, 3, and 4 showed high proportions of markers for which the difference between H_o_ and H_e_ was significantly different from 0, and only cluster 2 recorded a high proportion of markers with no significant differences (Table 5).

The analysis of molecular variance (AMOVA) revealed that the genetic variance was significantly different among and within clusters (phiPT = 0.61; *p* = 0.001). It was higher among clusters (1825.43) and lower (1152.90) within clusters (Table 6). The pairwise genetic distance among clusters was less than 0.50. Pairwise analysis using Nei’s genetic distance and fixation index (F_ST_) showed a low genetic distance and F_ST_ among clusters, and the values ranged from 0.24 to 0.61, and 0.20 to 0.42, respectively.

Genetic distance was highest between clusters 3 and 4 and lowest between clusters 3 and 2. Clusters 1 and 4 were 0.37 apart, clusters 2 and 4 were distant by 0.31. Genetic distances of 0.45 and 0.30 separated clusters 1 and 3, clusters 1 and 2, respectively. The F_ST_ was highest (0.42) between clusters 3 and 4 and lowest (0.20) between clusters 2 and 3. The genetic differentiation index between clusters 1 and 3 was 0.34, while it was 0.23 and 0.29 between the pairs (cluster 1, cluster 2) and (cluster 1, cluster 4), respectively (Table 6).

## 3. Discussion

### 3.1. Genetic Diversity of D. alata Accessions from CNRA Genebank Using Molecular Data

The knowledge of the genetic background within existing germplasm is important for proper conservation, management, and use for crop improvement through breeding [23]. Genetic diversity on yam has been dissected using phenotypic, molecular, or combined analyses [18]. The latter was advocated as a palliative method to limitations in using molecular markers or phenotypic descriptors alone in diversity analyses [18]. The application of molecular-based DNA markers in genetic characterization studies could allow a better understanding of the architecture and extent of plant species variability due to wide coverage of the genome and insensitivity to environmental and plant development stage influences as opposed to phenotypic data, which are limited in number and highly influenced by the plant growth phases and environment. In phenotypic assessment, some accessions often exhibit closely similar phenotypic traits although divergent at the molecular level. In addition, previous diversity studies on yam, particularly *D. alata*, were conducted using low throughput molecular markers, such as microsatellites or simple sequence repeats (SSR) [18,20,25,26,27,28,29,30], and other markers, such as AFLP and RAPD, with low reproducibility.

High throughput markers (SNPs) developed by the Diversity Array Technology (DArT) were used in this study to elucidate the structure and genetic diversity in the CNRA *D. alata* accessions from Côte d’Ivoire. Cluster analysis using the molecular-based SNP data categorized the 188 assessed accessions into four main genetic groups. Bayesian-based clustering method, kinship, and principal component analysis used as complementary approaches classified the accessions into four groups as well. The kinships and PCA showed higher relatedness of accessions within clusters. Shannon’s information index was low in general, but cluster 2, with the largest number of accessions, was more diverse than the others, showing that this cluster may be divided into several subgroups. These findings suggested that the accessions within clusters were genetically similar in their genome, and the current set of material could be considered highly valuable for genetic improvement in *D. alata*. Furthermore, a moderately low genetic diversity within the clusters could be used to form potential heterotic groups and new breeding populations by crossing individuals among the four clusters, thereby broadening the genetic base of the breeding programs [23,31].

However, analysis of molecular variance (AMOVA) showed that genetic differentiation within and among identified clusters was statistically significant. It was lower within clusters and higher among clusters. This is also confirmed by the low proportion of admixture in the present study. In this study, private alleles found only in one cluster associated with the high genetic variability among clusters could be interpreted as a lack of gene flow between accessions from different groups or that varietal groups are grown in geographically-distant areas with low seed exchanges among farmers. The low diversity within clusters could also mean that there are regional preferences for some dominant varieties, and thus farmers do not perceive the need to adopt several varieties simultaneously. This result contrasts findings by Agre et al. [18] in Benin, where high variability was observed within groups and areas as a reflection of diverse trait preferences by farmers and which could not be met in a single cultivar. It is noteworthy that this species is native to West Africa and, therefore, there are no wild relatives who can outcross with it, as is the case for *D. rotundata* [18], to increase variability within clusters. Indeed, historical events related to *D. alata* domestication revealed that this plant species was introduced to Africa from the southern border regions between Asia and Oceania in the 16th century, where natural outcrossing with its wild relatives, such as *D. nummularia,* was reported [4,32].

The Hardy–Weinberg equilibrium test indicated that most of the SNP markers used to assess the patterns of genetic diversity in *D. alata* accession showed significant differences between observed (H_o_) and expected (H_e_) heterozygosity not only in the total population but also in the four groups identified. The high difference between H_e_ and H_o_, as revealed by the lower negative value of inbreeding coefficient (Fis) in the population, would reflect an excess of heterozygous genotypes at the SNP loci analyzed [33,34]. This excess could mainly be attributed to the sexual mode of reproduction of this plant and to traditional seed management methods (accessions used in this study were collected in part from farmers). Indeed, yams are generally a dioecious and strictly cross-pollinated species, but vegetative propagation remains the main mode of propagation at the farmer level, while botanical seeds are mainly used for breeding purposes [32,35]. In the *D. alata* germplasm from Côte d’Ivoire, most of the accessions are landraces that rarely flower. When they flower, there are more male plants than females. In addition, flowering in female plants is sparse and irregular such that natural cross-pollination between accessions is a rare event [32]. It is also true that farmers conserve a part of the harvested tubers and use them as seeds in the next growing season [36]. The tubers of used accessions would then have been conserved and cultivated for generations as clones, resulting in the maintenance of a high rate of heterozygous genotypes in the germplasm. These observations are in agreement with those reported by Mignouna et al. [4], stating that farmers select genotypes that best suit their needs and advance them as separate cultivars. This partly explains the large number of traditional cultivars in West Africa. The presence of high proportions of major alleles in the population could, therefore, reflect the existence of varieties with producers’ desired traits [37]. In contrast to clusters 1, 3, and 4, the inbreeding coefficient was positive in cluster 2, which contained more accessions with homozygous genotypes. Many accessions in this cluster would be pure strains and could be potential sources of traits of interest in *D. alata* yam breeding and improvement programs.

### 3.2. Agronomic Trait Variations over Years for D. alata Accessions

The variability of quantitative agronomic traits for the 188 *D. alata* accessions was significantly different across years. The yield for vigorous plant rate (VPR) and medium vigorous plant rate (MVPR) was higher in 2018 compared to 2019. In 2018, planting was delayed (July) due to a lack of rainfall during the ideal planting periods (March, April, or May). This led to low disease severity compared to the year 2019. Egesi et al. [38] noted similar result trends. Indeed, these authors revealed that yams planted early in March are severely attacked by anthracnose disease than late plantations in April and May. Furthermore, late plantations in August showed much lower levels of anthracnose disease [38]. The low observed disease severity score might have positively influenced higher yields and more vigorous plants since anthracnose is the major biotic production-limiting factor worldwide of this yam species. However, Aighewi et al. [39] showed that late planting is not always the best option. They found that early plantations improved the yield of seed yam of *D. rotundata* accessions using minisett. The lower dry matter content observed in 2018 compared to 2019 could be due to a decrease in length of the growing season, resulting in shortened crop cycle by late rains (Supplementary Table S2). In Côte d’Ivoire, yam is usually harvested in December irrespective of the planting dates due to drought, which induces complete wilting of the plants at this period. Thus, tubers harvested in 2018 were not able to accumulate/bulk nutrients and minerals sufficiently to reach optimum dry matter content in a short time.

### 3.3. Agronomic Performance of D. alata Genotypes within and among Defined Clusters

In the present study, high variability among accessions or genotypes was observed for eight of the nine agronomic traits. These results indicate high variability in genotypes with diverse genetic backgrounds, which could lead to different agronomic performances across the years. This genetic variability presents opportunities for improving this yam species through breeding (use of the contrasting genotypes in hybridization) [32]. We also only noted high genotype × year interactions for five traits (anthracnose disease severity, tuber length, tuber width, tuber circumference, and weak vigorous plant rate), suggesting that the accessions were inconsistent/unstable in their performance; there were differences in ranking of the genotypes across years which could be the result of the rainfall variability across the two years. Thus, used traits were relevant for distinguishing accessions with the highest agronomic performance when tested across contrasting years within the same experimental site. When the traits, such as yield, vigorous plant rate, medium vigorous plant rate, and dry matter content, were tested, the genotype × year interactions were not significant, showing that these traits were not able to exhibit differences among accessions across the years in the study site. Furthermore, higher mean square values for all agronomic traits, in comparison to those of genotype × year interaction, could explain that the variance of these traits mainly depends on genetic control rather than the environment [40].

Generalized linear models or MANOVA performed with the nine quantitative agronomic traits of *D. alata* accessions indicated that the variability among molecular-based clusters only was highly significant among clusters defined by DAPC analysis. This finding would suggest a high variability among clusters at the agronomical level, which was in agreement with molecular results. However, phylogenic tree and DAPC analyses’ accession assignments only agreed for 25.41% accessions. The results indicated potential correlations between molecular data and agronomic traits in *D. alata* accessions, signifying that the relevant genomic regions involved in the variations of phenotypic traits expressions were efficiently detected by the SNP markers. In *D. alata* accessions, a low correlation between the genotypic and phenotypic distance matrices has also been observed [2]. However, Darkwa et al. [23] reported high and moderate correlations between the two data matrices in *D. rotundata* accessions with SNPs. The low correlation between the phenotypic and the genotypic data could have resulted from the natural and artificial selections on phenotypic variables, as these are under selection and influence of environmental factors. In contrast, the variation detected by molecular markers is commonly non-adaptive, and hence, not subject to natural and or artificial selections. This currently explains the interest in combining molecular and phenotypic analyses simultaneously instead of separating them to correct the level of mismatch observed in previous yam studies [18]. Phenotypic traits have the advantage of revealing the agronomic performance of a variety in a particular environment but have limited polymorphism, and they are subjected to changes in environmental conditions [2,18]. Harnessing the advantages of phenotypic and molecular markers improves the grouping of entries in a germplasm collection to provide valuable information for parental selection to realize and sustain genetic gain.

### 3.4. Broad-Sense Heritability for Agronomic Traits

Broad-sense heritability estimate in plant breeding is usually exploited for understanding the probability of inheriting target genes from parental lines after hybridization. It indicates the degree to which traits are expected to be inherited and provides an indication of the amount of genetic progress that would result in selecting the best individual based on the desired traits. Furthermore, the genotypic causes of genotype × year interaction are important indicators to estimate the variability of traits and make the promising genotypes possible to be selected based on the agronomic or phenotypic performance. Even though high broad-sense heritability was obtained for all agronomic traits despite the importance of genotype × year interaction, the environment was similar and estimation of the H^2^ based environment may have revealed the true value of the trait inheritance. Thus, plant breeders have a high probability of selecting promising genotypes for used traits when targeted in breeding programs [40,41].

## 4. Materials and Methods

### 4.1. Experimental Site

The experiment was conducted at the Research Station for Food Crops (SRCV) of the National Centre of Agronomic Research (CNRA) located in the Bouaké City, in central Côte d’Ivoire, at 7°44′ N latitude and 5°04′ W longitude. The Bouaké region constitutes a transition zone between the humid forests with short dry seasons and the dry savannas with long dry seasons [42]. Bouaké is characterized by a bimodal rainfall pattern with two rainy seasons, March to June and September to October, and two dry seasons, November to February and July to August [43]. The precipitation is irregular in the study site and often reaches an annual total of about 1100 mm, and the temperature fluctuates at around 28 °C, with variations of 3–5 °C. The soils at the research station are shallow, ferritic, and gravelly and derived from the alteration of granitic materials [44].

### 4.2. Plant Material and Experimental Design

A collection of 188 water yam accessions were selected from the core collection maintained at the National Centre of Agronomic Research (CNRA—Bouaké) and used for molecular-based diversity assessment. These accessions were partly the result of countrywide prospecting and collection missions carried out between 2008 and 2013 in major yam-producing areas of Southern, Central, and Northern Côte d’Ivoire. They were split into different variety groups, including landrace varieties (Betebete (56 accessions), Brazo (10 accessions), Douoble (16 accessions), Florido (41 accessions), and Nza (56 accessions)), and hybrid lines (57 accessions that are being evaluated for agronomic and culinary performance) (Supplementary Table S1).

For agronomic data collection, the experiments were carried out under field conditions on a plot of 60 m length and 42 m wide, i.e., a surface area of 0.56 ha. The field experiment comprised two completely randomized Fisher blocks with 2 m apart. In each block, the elementary plot containing an accession consisted of five plants at a spacing of 1 m between and within rows. After plowing and clearing the experimental plot, planting was conducted using either tuber fragments or whole tubers, and accessions were planted for two consecutive years, 2018 and 2019, at the beginning of the rainy season. Data displaying the meteorological variations during the two years of study is provided in Supplementary Table S2.

### 4.3. Phenotypic Data Collection

Nine most discriminative quantitative traits were selected and used to evaluate the agronomic performance of the 188 CNRA accessions. These traits were selected based on recommendations by Agre et al. [18]. The selected traits included tuber length, tuber width, tuber circumference, tuber yield, anthracnose disease severity, dry matter content, and plant vigor (visual assessment of the above-ground biomass of plants in a plot at two to five months after planting using a 1–3 assessment scale, where 1 = weak, 2 = moderate or medium, and 3 = vigorous), tuber length and width measurements were done using five tubers of each accession after harvesting, while the dry matter content was determined from 100 g of fresh flesh of two tubers dried at 70 °C for 72 h using an oven drying method. Anthracnose disease severity was first recorded two months after planting at the vegetative growth phase and then monthly until complete senescence of the plants using a 1–5 rating scale, as described by Kolade et al. [45] and Assala et al. [15].

### 4.4. Collection and Preparation of Leaf Samples of D. alata Accessions for Genotyping

Three (3) months after planting in 2018, young, healthy leaves of each of the 188 accessions were harvested in the morning hours and stored on dry ice. Ten (10) leaf discs of 5 mm diameter were sampled from the leaf blade of each accession using a biopsy curette. The leaf discs were then put into two 96-deep well PCR plates with the accession codes written on the wells of each plate. The plates were covered with silica gel and sent to Diversity Array Technology (DArT)^®^, Canberra, Australia, for DNA extraction, library construction, whole-genome resequencing, and SNP marker development.

### 4.5. Genotyping and SNP Marker Quality Control

Single row format data received from DArT were firstly converted into HapMap and VCF formats using KDcompute (https://kdcompute.seqart.net/kdcompute, accessed on 22 August 2021). The markers were first filtered based on the call rate of raw data [46,47]. SNP markers with a call rate ranging from 0.90 to 1 were selected for further analyses of quality control. The quality control implemented comprised removing markers with low minor allele frequencies (MAF < 0.05), genotype quality < 20, and read depth < 5.

### 4.6. Statistical Analyses

The structure and pattern of genetic diversity in *D. alata* accessions were assessed using the genotypic data generated with SNP markers. Clustering analysis of the 188 genotyped accessions was performed based on the Jaccard dissimilarity matrix and UPGMA method. A dendrogram was constructed for visualizing how closely accessions were related within each cluster using ape (analyses of phylogenetics and evolution) library package [48]. As complementary analysis based on kinship matrix, clustering-based Bayesian analysis and principal component analysis (PCA) were performed to efficiently determine the genetic relationships between accessions with FactoMineR [49] and FactoExtra R packages [50]. Discriminant analysis of principal component (DAPC) was also carried out using ‘genind objet’ and the find.clusters function in the adegenet package implemented in R software [21]. DAPC, based on the *k*-means clustering method, tends to reduce the variance among accessions within clusters and maximize the variance among clusters [21]. The Bayesian information criterion (BIC) was used to determine the optimum number of clusters to be retained and relevant in discriminating *D. alata* accessions. Following a cross-validation test using the ‘xval’ function, the optimum number of principal components (PCs) and discriminant functions to be retained and able to accurately predict the group membership was estimated with 100 repetitions, and the PCs retained was found to be associated with the lowest root mean square error (RMSE) [21,51,52]. A binary file was generated from the filtered VCF file and was then subjected to cross-validation approaches for population structure analysis. A cut-off value of 50% ancestry suggested through the Admixture analysis was used to estimate membership probabilities of the accessions for the groups identified [18,53]. Genetic diversity within and among groups using the SNP markers data was assessed through the number of different alleles (Na), observed heterozygosity (H_o_), expected unbiased heterozygosity (H_e_), Shannon information index (I), inbreeding coefficient index (Fis), polymorphism information content (PIC), and minor allele frequency (MAF). For each locus, the Hardy–Weinberg equilibrium test was assessed to compare the observed heterozygosity (H_o_) with the expected heterozygosity (H_e_) values. As the conditions of normality, independence, and heterogeneity of the residual values were not met for an analysis of variance (ANOVA), the Mann–Whitney *U* test was carried out to compare the mean ranks of H_o_ and H_e_ of each locus in each grouping pattern and the total population. The genetic differentiation among and within groups was estimated using the analysis of molecular variance (AMOVA), and the significance was tested with a non-parametric approach with 999 permutations. Differences between the groups defined were measured by computing PhiPT probability at the threshold of 0.05 and Nei’s genetic distance between paired groups [54]. GanAlEx software (version 6.503) was used to calculate genetic diversity parameters, Hardy–Weinberg equilibrium test, and Nei genetic distance, while IBM SPSS Statistics version 22 was used for the Mann–Whitney U test. The relationship between genotypic and phenotypic data was assessed by a non-parametric Mantel test based on Spearman’s rank correlations (rho) [23]. The correlation coefficients between the two data matrices and associated probabilities were computed with 9999 permutations using the vegan package of R version 4.1.1. Multiple analysis of variance (MANOVA) based on generalized linear models implemented in IBM SPSS Statistics was used to determine the variability of agronomic traits across years and to be able to differentiate between the grouping patterns identified based on molecular phylogenic analysis using the traits evaluated at the agronomic level. First, the normality of the residuals, homogeneity, and independence of variance was verified. The broad-sense heritability was then estimated using the following formula Equation (1):

(1) $$H^{2}=\frac{\sigma_{G}}{\sigma_{G}+\frac{\sigma_{P}}{r}}$$

where H^2^ is Broad sense heritability, _σG:_ genetic variance_, σP:_ Phenotypic variance, and r: Number of replications

## 5. Conclusions

The results from this study showed that *D. alata* accessions from CNRA genebank can be structured into four (4) main groups based on molecular SNP data. The genetic diversity within each cluster was relatively low. However, genetic variability among accessions was high among clusters. The genetic differentiation index (F_ST_) and Nei’s genetic distance between pairs of identified groups was low, while the FIS, which measures the difference between observed and expected heterozygosity, showed a very low inbreeding coefficient (Fis < 0) in the total population. This study provided reliable information and useful insights at molecular level into *D. alata* accessions from the CNRA genebank to guide plant breeding programs and sustainable management of existing genetic resources in Côte d’Ivoire. Furthermore, a highly significant variation in genotypes as revealed for all tested agronomic traits across the years allows the selection of promising parental genotypes for breeding activities to develop and supply more suitable varieties to farmers and other end-users in the region.

## Figures and Tables

**Table 1:** Variability in agronomic traits across two years based on the means ± standard deviation.

Agronomic Traits	Year of Assessment		Statistical Test	
	2018	2019	*F*	*p*-Value
Yield (t ha^−1^)	8.71 ± 5.19 ^a^	7.43 ± 3.46 ^b^	8.11	0.004
ADSS	2.43 ± 0.49 ^b^	3.08 ± 0.61 ^a^	118.22	<0.001
Tuber length (cm)	15.63 ± 3.71 ^b^	16.94 ± 4.22 ^a^	10.39	0.001
Tuber width (cm)	8.91 ± 1.65 ^b^	11.58 ± 2.58 ^a^	143.48	<0.001
Tuber circumference (cm)	21.80 ± 4.16 ^b^	23.58 ± 5.17 ^a^	14.27	<0.001
Dry matter content (%)	27.83 ± 3.27 ^b^	30.91 ± 4.82 ^a^	51.16	<0.001
VPR (%)	40.12 ± 26.78 ^a^	25.65 ± 26.75 ^b^	26.71	<0.001
MVPR (%)	51.64 ± 25.05 ^a^	37.76 ± 21.99 ^b^	29.74	<0.001
WVPR (%)	8.37 ± 15.58 ^b^	36.01 ± 27.40 ^a^	140.46	<0.001
Wilks’ lambda	-	-	0.175	<0.001

For a given parameter, mean values followed by the same letter are not statistically different at the threshold α = 0.05. ADSS: Anthracnose disease severity score; VPR: Vigorous plants rate; MVPR: Medium vigorous plant rate; and WVPR: Weak vigorous plant rate. “-”: not applied.

**Table 2:** Mean square and broad-sense heritability for agronomic traits of 188 *D. alata* accessions evaluated in Bouake for two years (2018 and 2019).

Agronomic Trait	Genotype	Year	Genotype × Year	H^2^
Yield (t ha^−1^)	6243.00 ***	573.00 ***	1042.00 ^ns^	0.72
ADSS	166.25 ***	70.42 ***	61.74 ***	0.74
Length (cm)	8696.00 ***	379.00 ***	2122.00 ***	0.56
Width (cm)	2368.60 ***	1389.70 ***	860.80 ***	0.79
Circumference (cm)	11,225.00 ***	728.00 ***	3484.00 ***	0.62
Dry matter content (%)	6945.00 ***	501.00 ***	1399.00 ^ns^	0.72
VPR (%)	296,970.00 **	28,334.00 ***	182,861.00 ^ns^	0.80
MVPR (%)	214,054.00 ^ns^	29,728.00 ***	164,661.00 ^ns^	0.71
WYPR (%)	214,404.00 ***	113,915.00 ***	106,119.00 *	0.74

ns; *; **; ****: not significant; significant at *p*-value thresholds of 0.05, 0.01, and 0.001, respectively; H^2^: Broad-sense heritability; ADSS: Anthracnose disease severity score; VPR: Vigorous plants rate; MVPR: Medium vigorous plant rate; and WVPR: Weak vigorous plant rate.

**Table 3:** Agronomic performance of clusters defined by DAPC analysis based on the mean ± standard deviation of nine quantitative traits.

Agronomic Traits	Cluster 1 (*n* = 41)	Cluster 2 (*n* = 69)	Cluster 3 (*n* = 42)	Cluster 4 (*n* = 36)	CV (%)	Statistical Tests	
						F	*p*
Yield (t ha^−1^)	7.17 ± 4.53 ^a^	6.47 ± 3.72 ^ab^	5.98 ± 3.71 ^b^	6.26 ± 3.65 ^b^	60.21	2.80	0.039
ADSS	2.77 ± 0.70 ^ab^	2.71 ± 0.72 ^b^	2.91 ± 0.73 ^a^	2.65 ± 0.62 ^b^	25.53	3.95	0.008
Length (cm)	17.88 ± 4.87 ^a^	15.82 ± 3.97 ^b^	15.47 ± 4.42 ^b^	16.06 ± 4.27 ^b^	26.74	9.54	<0.001
Width (cm)	10.47 ± 2.78 ^a^	10.60 ± 2.86 ^a^	9.77 ± 3.01 ^b^	10.05 ± 2.66 ^ab^	27.69	3.27	0.021
Circumference (cm)	23.25 ± 5.04 ^a^	23.32 ± 5.32 ^a^	21.38 ± 5.32 ^b^	22.47 ± 5.39 ^ab^	23.26	5.15	0.001
DMC (%)	28.65 ± 3.88 ^a^	28.89 ± 3.84 ^a^	27.49 ± 4.11 ^b^	29.25 ± 3.74 ^a^	13.61	6.18	<0.001
VPR (%)	36.48 ± 36.92	32.28 ± 35.66	28.22 ± 34.14	32.84 ± 36.48	110.44	1.40	0.239
MVPR (%)	45.40 ± 34.22	48.92 ± 33.67	43.65 ± 34.10	45.29 ± 34.88	73.72	0.90	0.438
WYPR (%)	18.11 ± 26.23 ^b^	19.06 ± 27.01 ^b^	28.02 ± 33.20 ^a^	21.86 ± 29.12 ^ab^	134.28	4.09	0.007
Wilks’ lambda						2.93	<0.001

For a given parameter, mean values followed by the same letter are not statistically different at the threshold α = 0.05. ADSS: Anthracnose disease severity score; VPR: Vigorous plant rate; MVPR: Medium vigorous plant rate; WVPR: Weak vigorous plant rate.

**Table 4:** Genetic diversity parameters for 188 *D. alata* accessions and within the four distinct clusters based on 11,722 SNP markers data.

Grouping Pattern	Na	A^P^	I	MVF	H_o_	H_e_	Fis	PIC	MAF
Population (*n* = 188)	23,444	0.00	0.34	1.63 × 10^−5^	0.294	0.158	−0.398	0.276	0.209
Cluster 4 (*n* = 38)	21,669	0.00	0.25	0.004	0.185	0.096	−0.927	0.154	0.093
Cluster 3 (*n* = 43)	21,798	0.00	0.28	0.007	0.211	0.138	−0.529	0.183	0.115
Cluster 1 (*n* = 26)	21,153	0.00	0.29	0.008	0.227	0.121	−0.876	0.184	0.116
Cluster 2 (*n* = 81)	23,442	175.00	0.54	0.022	0.259	0.278	0.068	0.270	0.202

*n*: Number of accessions; Na: Number of different allele at each locus; I: Shannon information index; H_o_: observed heterozygosity; H_e_: expected heterozygosity; Fis: Inbreeding coefficient, A^P^: Number of private alleles, PIC: polymorphism information content; and MAF: minor allele frequencies; MDF: missing value frequencies.

**Table 5:** Hardy–Weinberg equilibrium and Mann–Whitney U test for detecting differences between H_o_ and H_e_ among *D. alata* accessions.

Hardy–Weinberg Equilibrium Test			Mann–Whitney U Test			
Clusters	Proportion (%) of SNP Markers		Mean Rank		Statistical Tests	
	Significant	No Significant	H_o_	H_e_	*U* × 10^5^	*p*
Cluster 4	62.81	37.19	3346.40	3249.24	52.08	<0.037
Cluster 3	70.54	29.46	3543.78	2792.65	35.16	<0.001
Cluster 1	66.67	33.33	3442.60	2724.33	34.58	<0.001
Cluster 2	48.71	51.29	3857.80	4617.80	73.75	<0.001
Population	62.18	37.82	5013.12	3517.88	59.065	<0.001

H_o_: Observed heterozygosity; H_e_: Expected heterozygosity.

**Table 6:** Nei’s genetic distance, fixation index (F_ST_), and analysis of molecular variance (AMOVA) among 188 *D. alata* accessions.

Nei’s Genetic Distance and Fixation Index (F_ST_)			Analysis of Molecular Variance (AMOVA)						
Clusters	Nei’s Distance	F_ST_	Sources		df	SS	MS	Variance	Variance %
3-4	0.61	0.42	Among clusters		3	239,269.83	79,756.61	1825.43	61.29
1-4	0.37	0.29	Within clusters		184	212,132.69	1152.90	1152.90	38.71
2-4	0.31	0.24	Statistical tests	phiPT				0.61	
1-3	0.45	0.34		P				0.001	
2-3	0.24	0.20							
2-1	0.30	0.23							

1: cluster 1, 2: cluster 2, 3: cluster 3, 4: cluster 4; df: degrees of freedom; SS: Sum of Squares; MS: Mean Squares.

## Data Availability

Most of the data are contained within the article and supplementary files. Additional data are available on request from the corresponding author.

## Supplementary Materials

The following materials are available online at https://www.mdpi.com/article/10.3390/plants10122562/s1, Table S1. List of accessions and variety groups evaluated in the study; Table S2. Weather parameters during observation periods in Bouaké, Côte d’Ivoire; Figure S1. Bayesian information criteria (BIC) showing the optimum number of clusters; Figure S2. Grouping pattern of 188 CNRA *D. alata* accessions at different K levels: (A) K = 2, (B) K = 3, (C) K = 5, (D) K = 6, (E) K = 7, (F) K = 8 based on the Bayesian clustering method.
