# Supplementary material for: Diversity of Water Yam (Dioscorea alata L.) Accessions from Côte d’Ivoire Based on SNP Markers and Agronomic Traits
Source: Plants (Basel). 2021 Nov 24;10(12):2562. doi: 10.3390/plants10122562 (PMC8705775; doi:10.3390/plants10122562)
Supplement: Supplementary file 1 [file plants-10-02562-s001.zip › Supplementary Figure all.pdf]

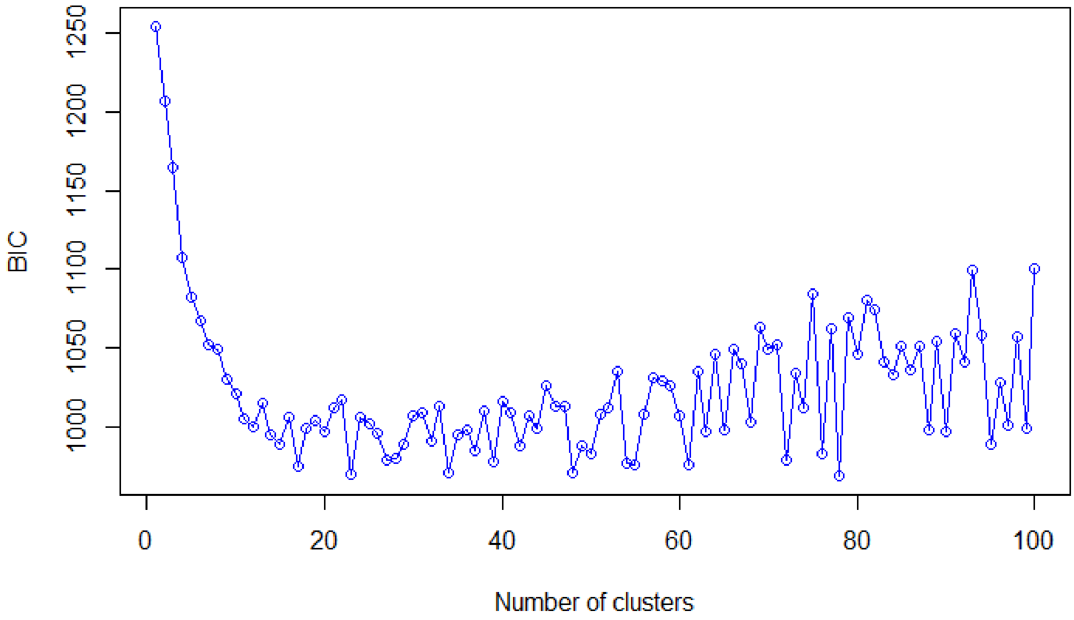

Figure S1. Bayesian information criteria (BIC) showing the optimum number of clusters.

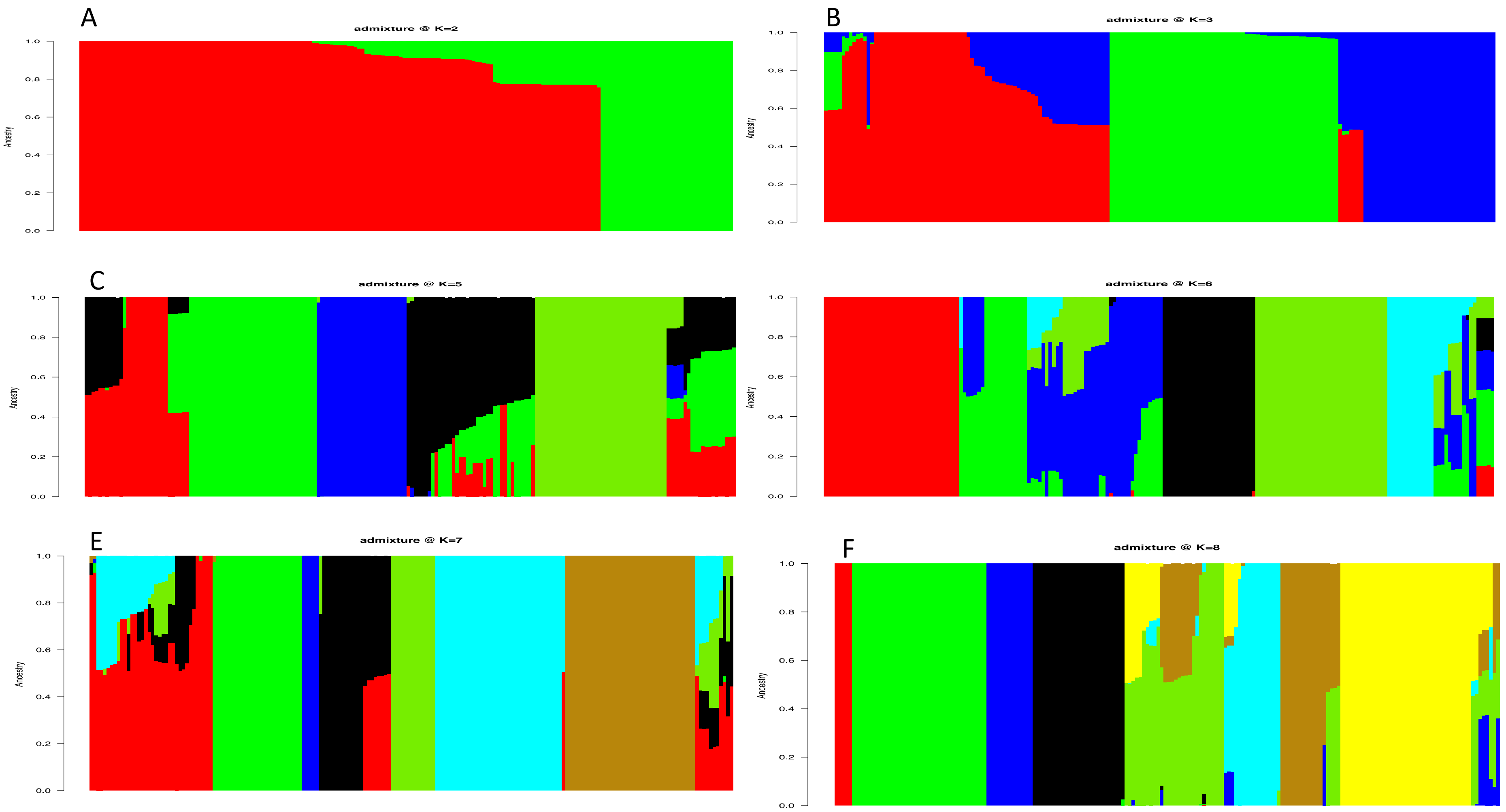

Figure S2. Grouping pattern of 188 CNRA *D. alata* accessions at different K levels: (A) K=2, (B) K=3, (C)K=5, (D) K=6, (E) K=7, (F) *K* = 8 based on the Bayesian clustering method. The colour displays different clusters. Each vertical bar corresponds to an accession and colour proportion in each bar represents the probability of each accession to be affiliated to the different clusters.
