# Supplementary material for: Diversity of Water Yam (Dioscorea alata L.) Accessions from Côte d’Ivoire Based on SNP Markers and Agronomic Traits
Source: Plants (Basel). 2021 Nov 24;10(12):2562. doi: 10.3390/plants10122562 (PMC8705775; doi:10.3390/plants10122562)
Supplement: Supplementary file 1 [file plants-10-02562-s001.zip › Table S1.pdf]

Table S1: List of accessions and variety groups evaluated in the study.

| Accessions | VRG | Accessions | VRG | Accessions | VRG | Accessions | VRG |
|---|---|---|---|---|---|---|---|
| civcda 002 | Brazo | civcda 086 | Betebete | civcda 354 | Betebete | civcda 375 | Betebete |
| civcda 022 | Florido | civcda 088 | Betebete | civcda 359 | nza | civcda 376 | Florido |
| civcda 035 | Douoble | civcda 101 | Betebete | civcda 367 | nza | civcda 383 | Douoble |
| civcda 050 | Brazo | civcda 105 | Florido | civcda 368 | Florido | civcda 388 | nza |
| civcda 053 | Betebete | civcda 109 | Florido | civcda 373 | Betebete | civcda 393 | nza |
| civcda 059 | Brazo | civcda 110 | Brazo | civcda 377 | Betebete | civcda 010 | Brazo |
| civcda 061 | Brazo | civcda 115 | Hybrid | civcda 378 | Florido | civcda 011 | Florido |
| civcda 062 | Brazo | civcda 119 | Hybrid | civcda 387 | Betebete | civcda 030 | Florido |
| civcda 100 | Douoble | civcda 122 | Hybrid | civcda 389 | Douoble | civcda 036 | Florido |
| civcda 114 | Hybrid | civcda 125 | Hybrid | civcda 390 | nza | civcda 038 | Florido |
| civcda 121 | Hybrid | civcda 126 | Hybrid | civcda 442 | Florido | civcda 047 | Florido |
| civcda 130 | Hybrid | civcda 127 | Hybrid | civcda 445 | Betebete | civcda 065 | Florido |
| civcda 133 | Hybrid | civcda 132 | Hybrid | civcda 008 | Brazo | civcda 068 | Betebete |
| civcda 142 | Hybrid | civcda 140 | Hybrid | civcda 009 | Douoble | civcda 111 | Douoble |
| civcda 146 | Hybrid | civcda 144 | Hybrid | civcda 015 | Florido | civcda 112 | Betebete |
| civcda 148 | Hybrid | civcda 151 | Hybrid | civcda 044 | Betebete | civcda 116 | Hybrid |
| civcda 153 | Hybrid | civcda 155 | Hybrid | civcda 060 | Douoble | civcda 134 | Hybrid |
| civcda 158 | Hybrid | civcda 157 | Hybrid | civcda 063 | Florido | civcda 135 | Hybrid |
| civcda 162 | Hybrid | civcda 160 | Hybrid | civcda 066 | Florido | civcda 136 | Hybrid |
| civcda 166 | Hybrid | civcda 163 | Hybrid | civcda 083 | Betebete | civcda 138 | Hybrid |
| civcda 167 | Hybrid | civcda 168 | Hybrid | civcda 106 | Douoble | civcda 141 | Hybrid |
| civcda 207 | Betebete | civcda 172 | Hybrid | civcda 113 | Douoble | civcda 145 | Hybrid |
| civcda 214 | Douoble | civcda 175 | Hybrid | civcda 117 | Hybrid | civcda 147 | Hybrid |
| civcda 223 | Betebete | civcda 179 | Hybrid | civcda 129 | Hybrid | civcda 150 | Hybrid |
| civcda 229 | nza | civcda 180 | Hybrid | civcda 137 | Hybrid | civcda 156 | Hybrid |
| civcda 232 | Betebete | civcda 187 | Hybrid | civcda 149 | Hybrid | civcda 178 | Hybrid |
| civcda 307 | Douoble | civcda 189 | Hybrid | civcda 152 | Hybrid | civcda 202 | Florido |
| civcda 310 | nza | civcda 191 | Hybrid | civcda 165 | Hybrid | civcda 217 | Betebete |
| civcda 320 | Florido | civcda 195 | Hybrid | civcda 170 | Hybrid | civcda 218 | Douoble |
| civcda 331 | Douoble | civcda 205 | Florido | civcda 174 | Hybrid | civcda 245 | Betebete |
| civcda 332 | Florido | civcda 208 | nza | civcda 184 | Hybrid | civcda 266 | nza |
| civcda 333 | Florido | civcda 209 | nza | civcda 186 | Hybrid | civcda 305 | Betebete |
| civcda 339 | Douoble | civcda 216 | Betebete | civcda 192 | Hybrid | civcda 327 | Betebete |
| civcda 341 | Betebete | civcda 224 | Florido | civcda 235 | Douoble | civcda 349 | nza |
| civcda 347 | Betebete | civcda 228 | Florido | civcda 236 | Douoble | civcda 355 | Florido |
| civcda 352 | Betebete | civcda 244 | Betebete | civcda 241 | Betebete | civcda 358 | nza |
| civcda 361 | nza | civcda 253 | Betebete | civcda 252 | Betebete | civcda 364 | nza |
| civcda 363 | Florido | civcda 259 | Betebete | civcda 268 | Betebete | civcda 369 | Florido |
| civcda 366 | Betebete | civcda 272 | Betebete | civcda 275 | Betebete | civcda 386 | Betebete |
| civcda 441 | nza | civcda 311 | nza | civcda 314 | Florido | civcda 446 | Florido |
| civcda 443 | Betebete | civcda 316 | Florido | civcda 322 | nza | civcda 447 | Hybrid |

| Accessions | VRG | Accessions | VRG | Accessions | VRG |
|---|---|---|---|---|---|
| civcda 014 | Brazo | civcda 317 | Betebete | civcda 329 | Betebete |
| civcda 026 | Betebete | civcda 325 | nza | civcda 343 | nza |
| civcda 039 | Florido | civcda 326 | Betebete | civcda 346 | Betebete |
| civcda 040 | Florido | civcda 337 | nza | civcda 350 | Betebete |
| civcda 041 | Florido | civcda 344 | Betebete | civcda 351 | Florido |
| civcda 046 | Betebete | civcda 345 | Florido | civcda 365 | Betebete |
| civcda 064 | Florido | civcda 348 | Betebete | civcda 370 | Betebete |
| civcda 080 | Betebete | civcda 353 | Florido | civcda 374 | Betebete |

VRG Varietal group
