# Supplementary material for: Diversity of Water Yam (Dioscorea alata L.) Accessions from Côte d’Ivoire Based on SNP Markers and Agronomic Traits
Source: Plants (Basel). 2021 Nov 24;10(12):2562. doi: 10.3390/plants10122562 (PMC8705775; doi:10.3390/plants10122562)
Supplement: Supplementary file 1 [file plants-10-02562-s001.zip › Table S2.pdf]

Table S2. Weather parameters during observation periods in Bouaké, Côte d'Ivoire

Observation Periods	Weather Parameters	Year	
		**2018**	**2019**
2 months after planting (P1)	Rainfall (mm)	234.7	253.6
	Number of rainy days	13	7
	Temperature (°C)	24.1	23.7
	Wind speed (km/h)	9	14
	Relative humidity (%)	93	88
3 months after planting (P2)	Rainfall (mm)	82	116.1
	Number of rainy days	8	9
	Temperature (°C)	24.3	24.1
	Wind speed (km/h)	8	10
	Relative humidity (%)	82	87
4 months after planting (P3)	Rainfall (mm)	0	147.7
	Number of rainy days	0	10
	Temperature (°C)	26.2	24.3
	Wind speed (km/h)	8	10
	Relative humidity (%)	73	85

Source: GDSME, CNRA and www.historique-meteo.net
